# Manatees display diel trends in acoustic activity at two microhabitats in Belize

**DOI:** 10.1371/journal.pone.0294600

**Published:** 2023-11-17

**Authors:** Beth Brady, Carly Sarbacker, Jake Andrew Lasala, Maria Maust-Mohl, Kristi Ashley Collom, Linda Searle, Laura J. May-Collado, Eric Angel Ramos

**Affiliations:** 1 Mote Marine Laboratory, Sarasota, Florida, United States of America; 2 Department of Biology, University of Vermont, Burlington, Vermont, United States of America; 3 Department of Psychology, Manhattan College, Riverdale, New York, United States of America; 4 Department of Psychology, Hunter College, City University of New York, New York, New York, United States of America; 5 ECOMAR, Belize City, Belize; 6 Smithsonian Tropical Research Institute Apartado Postal 0843–03092 Panamá, Panama, República de Panamá; 7 Fundación Internacional para la Naturaleza y la Sustentabilidad (FINS), Chetumal, Quintana Roo, México; Annamalai University, INDIA

## Abstract

Many marine mammals exhibit diel trends in vocal production, which can provide information on habitat use and behavioral activity. In Belize, Antillean manatees (*Trichechus manatus manatus*) commonly inhabit small depressions in the substrate or deep-water coves known as “resting holes”. Determining if manatees exhibit diel temporal trends in their call production rate and call types between microhabitats can provide insights into their diurnal and nocturnal activity patterns. Here, we investigate the diel vocalization patterns of wild Antillean manatees in two adjacent resting holes off of St. George’s Caye, Belize. Recordings of manatees were made using a bottom-mounted hydrophone located near a reef barrier reef for nine days in July of 2017 and ten days in January of 2018. To explore if and how manatee acoustic activity differs between sites, we compared the number of calls per hour, the number of manatee positive hours, the number of tonal and atonal sounds, and the number of boats detected across sites. A total of 370 hours of acoustic recordings were analyzed resulting in the detection of 3,262 calls. There were no significant differences in the number of manatee calls produced per hour between sites. The average number of calls produced by manatees decreased over the course of several days. The proportion of tonal calls decreased with hours after sunset and increased in boat presence. These results suggest manatees in this region may exhibit different diel activity patterns which appear to be influenced by the characteristics of the environment. These findings can support ongoing conservation and management efforts to safeguard species in Belize.

## Introduction

Passive acoustic monitoring (PAM) techniques offer a powerful means of studying marine mammals by providing cost-effective and minimally invasive, long duration recordings in remote locations. Moreover, they are particularly useful for examining activity when animals are not easily observed during non-daylight hours. Many marine mammals exhibit diel patterns in vocal activity in relation to feeding (e.g., blue whales (*Balaenoptera musculus*) [1]; melon headed whales (*Peponocephala electra*) [2]), mating (e.g., North Atlantic right whales (*Eubalaena glacialis*) [3]; harbor seals (*Phoca vitulin*a) [4]; humpback whales (*Megaptera novaeanglia*e) [5]), and habitat use (e.g., Indo-Pacific humpback dolphin (*Sousa chinensis*) [6]; North Atlantic minke whales (*Balaenoptera acutorostrata*) [7]). By examining diel patterns in vocal behavior, researchers can gain insights on how animals utilize their space at various spatial and temporal scales ultimately informing conservation and mitigation efforts at the species level by identifying critical habitats (e.g. North Atlantic right whales [8]: blue whales [9]).

The West Indian manatee (*Trichechus manatus*) faces significant challenges in its freshwater and marine habitats throughout its distribution. Declining populations suffer from high mortality rates due to watercraft collisions and habitat loss resulting from development [10]. Passive acoustic monitoring techniques can help bridge the gap in our understanding of manatee habitat use in turbid aquatic habitats by detecting their vocal activity [11,12]. Longitudinal records of underwater soundscapes can also reveal associations between manatee presence, ambient noise levels [13,14], and vessel-generated noise [15–17].

Antillean manatees (*T*. *m*. *manatus*) inhabit diverse habitats throughout the Western Caribbean Sea, where their temporal activity patterns are not well understood [18]. Although manatees lack pineal glands, which regulate circadian rhythms [19], some studies suggest that Antillean manatees in some areas might have shifted their diel activities to rest primarily in daytime hours and forage in crepuscular and nocturnal hours to reduce the likelihood of impacts from humans from boats and hunting pressure [20–23]. For example, diel patterns of activity detected from VHF tagged manatees in Tabasco, Mexico indicated resting behavior in the afternoon and foraging from 1800 h to 0000 h [24]. In Nicaragua and Costa Rica, interviews with fisherman reported manatees displayed diurnal/nocturnal patterns of activity involving daytime rest in deep, quiet waters, and leaving to feed in the afternoon to early morning [21,25]. These studies independently show that diel activity patterns of manatees can be strongly influenced by human activities within a relatively short time.

In coastal Belize, manatees frequent areas locally known as "resting holes" [22,26]. These resting holes are relatively deep depressions in the coastal marine substrate that are typically calm and protected from wave action and weather conditions as they are located at the end of channels, in lagoons, or coves [22]. Visual observations of daily activity patterns of manatees at twelve resting holes in Belize suggested they are primarily used during the day and sometimes at night [22]. However, these surveys only lasted until midnight and may not fully represent the nocturnal activity of manatees. Acoustic surveys can help fill this gap by detecting manatee presence during non-daylight hours.

Antillean manatees rely on sound to communicate [27–29]. Like most mammals, manatees vocalizations are produced by forcing air through their vocal folds [30]. These vocalizations are short duration (<1 s) and often classified as tonal harmonic sounds (squeaks) and atonal sounds (squeals and screeches) [28,31]. These vocalizations serve a range of communication purposes, especially between mothers and calves [28,32–35] and during social interactions [33,36,37], making manatees excellent candidates for passive acoustic monitoring.

In this study, we investigated the diel vocal activity of wild Antillean manatees in two adjacent habitats near St. George’s Caye in Belize. We deployed a bottom-mounted underwater recorder to capture manatee vocalizations to determine if manatees show variation in their vocal activity throughout the day, and to evaluate if differences in habitat (e.g. boat presence) influence the patterns observed.

## Methods

### Data collection

#### Study site

**Fig 1:**
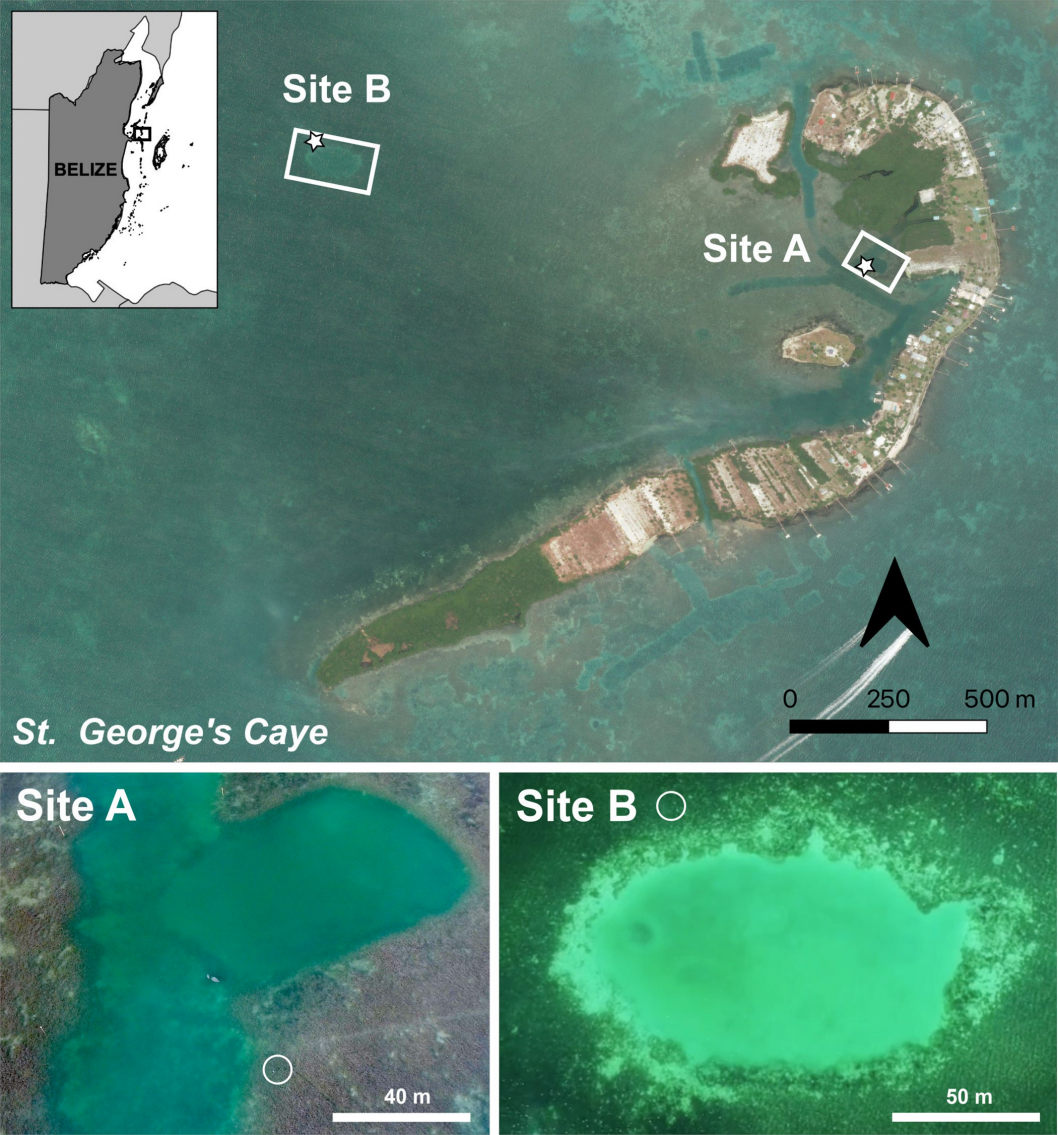
Map of the study area at St. George’s Caye in Belize and images of Site A (top right) and Site B (top left). The SoundTrap HF300 was deployed in a seagrass channel near the resting hole for Site A (white circle), a man-made dredged channel, and on seagrass flats near a resting hole for Site B (white circle). The basemap in the top map was generated with drone-based orthomosaic images of St. George’s Caye, gathered and constructed by Eric A. Ramos.

Data for this study were gathered on the leeward side of St. George’s Caye, which is a small crescent-shaped island located 9.5 km east of mainland Belize near the Belize Barrier Reef (Fig 1). The caye is surrounded by expansive seagrass flats, sand patches, deep channels, and holes, and is regularly inhabited by manatees of all age and sex classes [38]. Our recordings were collected passively, without approaching manatees, as part of ongoing work by ECOMAR at the caye. Research was approved by the Belize Fisheries Department and Belize Forest Department.

Manatee vocalizations were recorded near two resting holes in the area (Fig 1). Site A was at the end of a dredged channel adjacent to a deep rectangular-shaped depression used as a resting hole (4 m deep). This site was created by dredging (area: 1,806 m^2^) a section of the eastern edge of St. George’s Caye. Site B, approximately 1 km northeast from Site A, was near a much larger oval-shaped depression used as a resting hole (area: 8,844 m^2^). The depression consisted of a silt and sandy bottom and depths of 2–5 m, surrounded by seagrass flats. Site B overlapped with a common route for boats traveling through this area of the coast of Belize, whereas boats at Site A were restricted to several small vessels owned by local fishers. Both habitats were surrounded by contiguous seagrass flats composed primarily of turtle grass (*Thalassia testudinum*), manatee grass (*Syringodium filiforme*), and shoal grass (*Halodule wrightii*), interspersed with various species of algae and patches of sand.

### Acoustic recordings

Two calibrated SoundTrap 300 HF (Ocean Instruments, New Zealand) systems (flat frequency response: 20 Hz to 100 kHz [± 2 dB], clip level: 172 dB re 1μPa) were used to continuously record ambient noise and manatee vocalizations at each site. The date and time were embedded in the file name of each wav file. Recordings from Site A (sample rate 576 kHz, 16-bit resolution) were obtained from July 7–10 and July 13–16, 2017 (Fig 1). Recordings from Site B (sample rate 96 kHz, 16-bit resolution) were obtained from January 10–20, 2018 (Fig 1). At each location, the SoundTrap was suspended by a rope in the water column to 1–1.5 m in depth and was anchored to the seafloor with a cinderblock.

### Data analysis

#### Acoustic analysis

**Fig 2:**
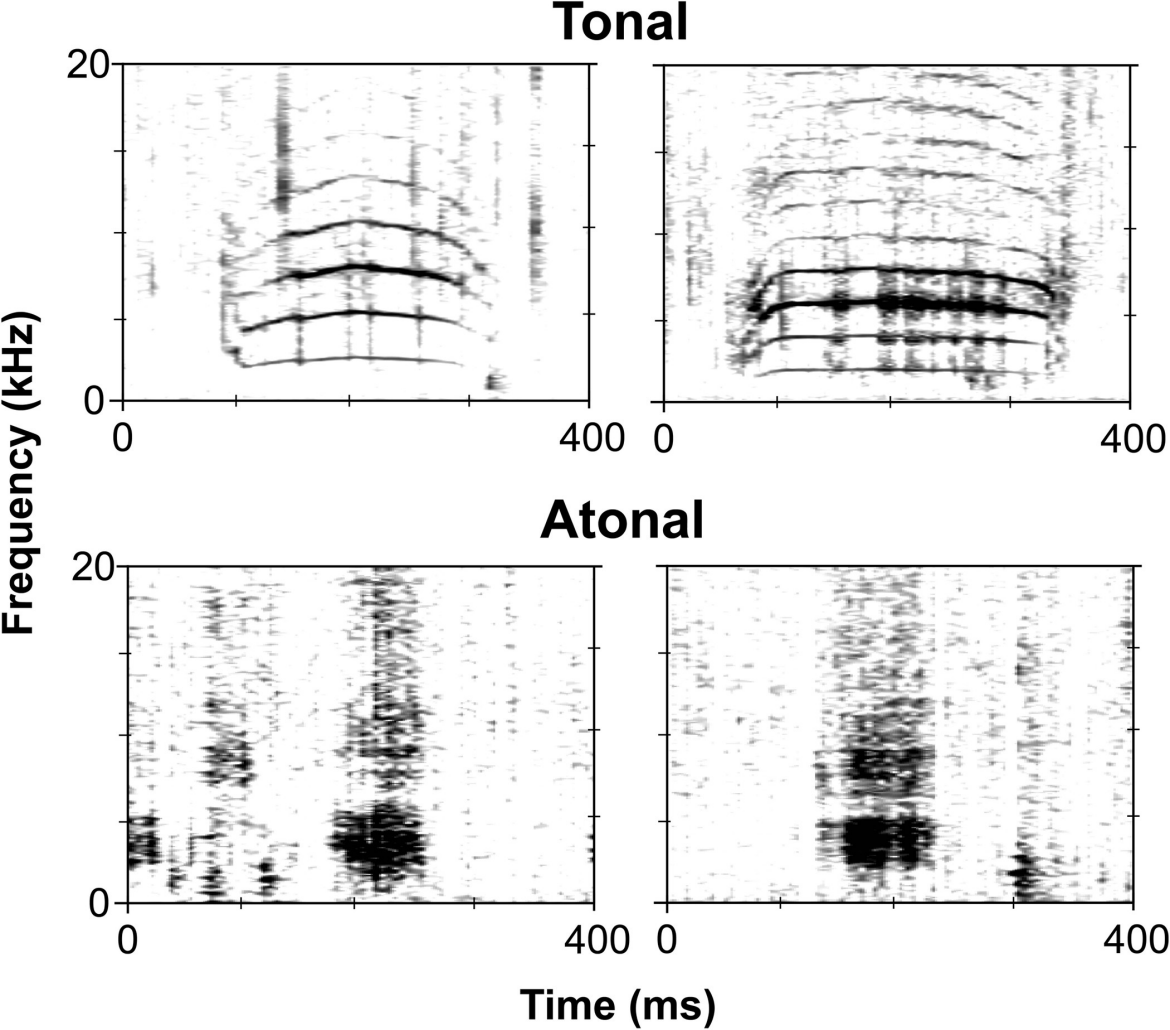
Spectrograms of wild Antillean manatee vocalizations recorded near two resting holes during this study. The top spectrograms show tonal vocalizations and the bottom two spectrograms show atonal vocalizations. Spectrogram parameters: Hanning window; 90% overlap; 512 FFT.

Acoustic files were visualized in Raven Pro 1.5 and 1.6 [39]. Recordings from Site A were downsampled to 96 kHz before viewing. Spectrograms of recordings from each site were viewed with a fast Fourier transform (FFT) (DFT: 2,048 points; Hann window; overlap: 90%; hop size: 1.07 samples; time resolution: 10.7 ms) to identify manatee calls. Manatee calls were distinguished from the sounds of bottlenose dolphins (the only other marine mammal in the region producing vocalizations of similar structure) using previously reported characteristics of manatee calls [31]. Manatee calls were manually detected by seven trained observers. Detected calls from the observers were then verified by two researchers skilled in identifying manatee vocalizations. Most calls had a poor signal to noise ratio or only had one harmonic band which made it difficult to reliably classify sounds by call type. For this reason, each verified call was broadly classified as: 1) tonal, which lacks deterministic chaos, or 2) atonal, which contains deterministic chaos and lack of inflection points [14] (Fig 2). Both researchers had to agree on classification. If they disagreed, a third skilled reviewer made the final decision regarding classification of the call as tonal or atonal. All verified calls detected, regardless of SNR, were included in the analysis.

#### Boat presence

Manatees are known to decrease vocalization rates during feeding and social behaviors in the presence of elevated noise levels from boats [14]. We recorded the number of boat detections per hour to determine if boat presence influenced the detection of manatee vocalizations. All observations of boat noise were manually detected by one skilled observer in spectrograms generated in Raven 1.6. Boat noise was determined by auscultation as well as long duration (10–200 sec) of broadband noise with most of the energy content <1000 Hz.

#### Manatee diel call detection

We tallied the number of manatee calls detected per clock hour for each site. Since recordings were not simultaneous and recorded during different times of year, we could not assess use of both habitats between seasons. In addition, due to the close proximity between the resting holes (~ 1 km), we statistically tested for differences between sites. If there were no differences in detection, the data from both holes were pooled together. Data were tested for normality (Shapiro-Wilk) and homogeneity of variance.

Following the methodology of Cascao et al. (2020) [40], we calculated manatee positive hours (MPH). MPH is the proportion of the number of hours with at least one detection of a call over the total number of hours for which recordings were made per day. A Welch’s two sample t-test was run to compare MPH between the two sites. The dates of sampling were inconsistent time frames, so the variable of sampling *day* was added to create an unbiased comparison. An analysis of variance (ANOVA) was run to determine if there was a significant difference in MPH due to the day or the number of boats.

A binomial generalized additive mixed model (GAMM, logit link function) was used to assess whether the presence or absence of calls was significantly different due to time (hour: smoothed), day, and the number of boats. Hour after sunset was added as an explanatory variable to account for the differences in sunlight due to the different seasons (July of 2017 and January of 2018). Because sunset varies between the two timeframes, *hour after sunset* was calculated for each site to account for variation in daylight hours. Hour after sunset was obtained from the National Oceanic and Atmospheric Administration’s Global Monitoring Laboratory’s Solar Calculator [41].

#### Factors influencing manatee call detection

A generalized linear model (GLM) was run to identify if the number of calls were significantly different due to time of day (hour) and the number of boats. One of the most common vocalizations produced by manatees are tonal sounds [14,31,37]. Here we used a beta regression generalized additive model (GAM, logit link function) to determine whether the proportion of tonal calls was significantly different due to hour after sunset (explanatory: smoothed), day, and the number of boats. Models were assessed using a stepwise selection method by comparing Akaike Information Criterion (AIC) values. All analyses were completed in R [42], including the use of packages *mgcv*, *effectsize*, *ggpubr*, *ggplot2*, and *visreg* [43–47].

## Results

### Calls detected

**Fig 3:**
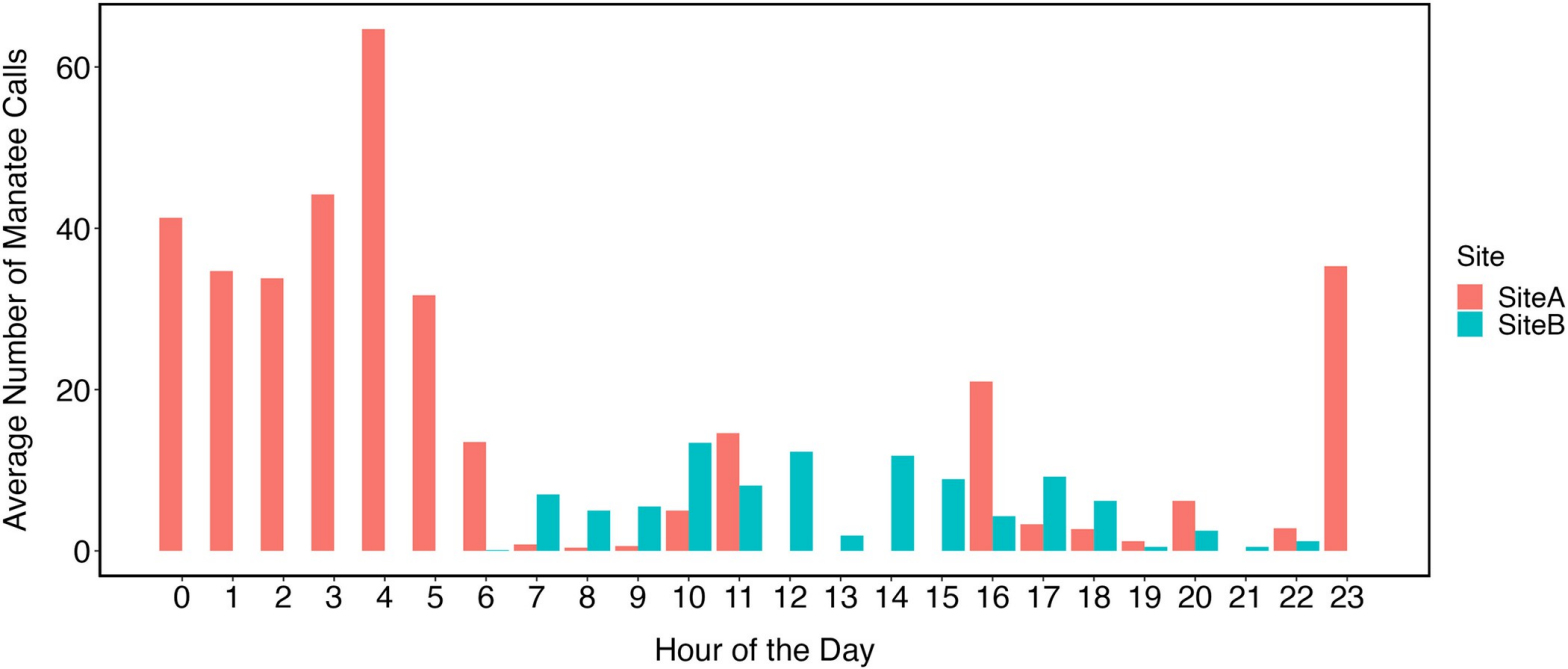
Bar graph depicting the number of Antillean manatee vocalizations detected during each recording hour at the recording sites (Total of 370 hr recordings from three deployments of the SoundTrap HF300).

Overall, we detected 3,262 wild manatee vocalizations from 370 total hours of recordings. At Site A (133 hours of recordings), we detected 2,217 calls of which 1,884 calls were classified as tonal and 333 calls were atonal. At Site B (237 hours of recordings), we detected 1,045 calls of which 856 calls were classified as tonal and 88 calls were atonal. Fig 3 shows the vocalization rate (average number of detected calls per hour of deployment) across all recording days for each site.

### Boat presence

**Fig 4:**
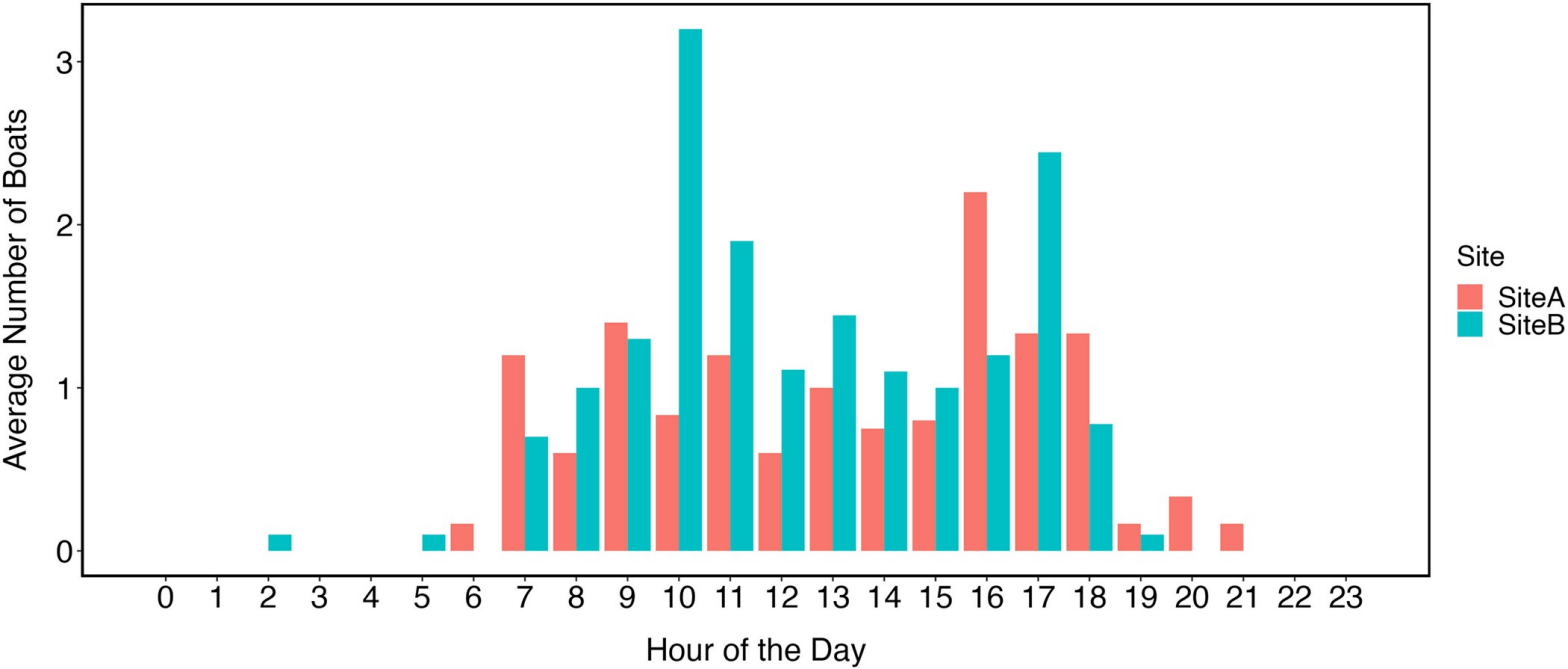
Average number of boats detected per hour at each recording site.

At Site A, we detected an average of 0.55 boats/hr (range: 0–4 boats/hour) with all detections occurring between 06:00 h and 21:00 h (Fig 3). At Site B, we detected an average of 0.72 boats/hr (range: 0–7 boats/hour) with all but two detections occurring between 06:00 h and 19:00 h. In both locations, the majority of boat detections occurred between 08:00 h and 17:00 h (Fig 4).

### Diel occurrence

**Fig 5:**
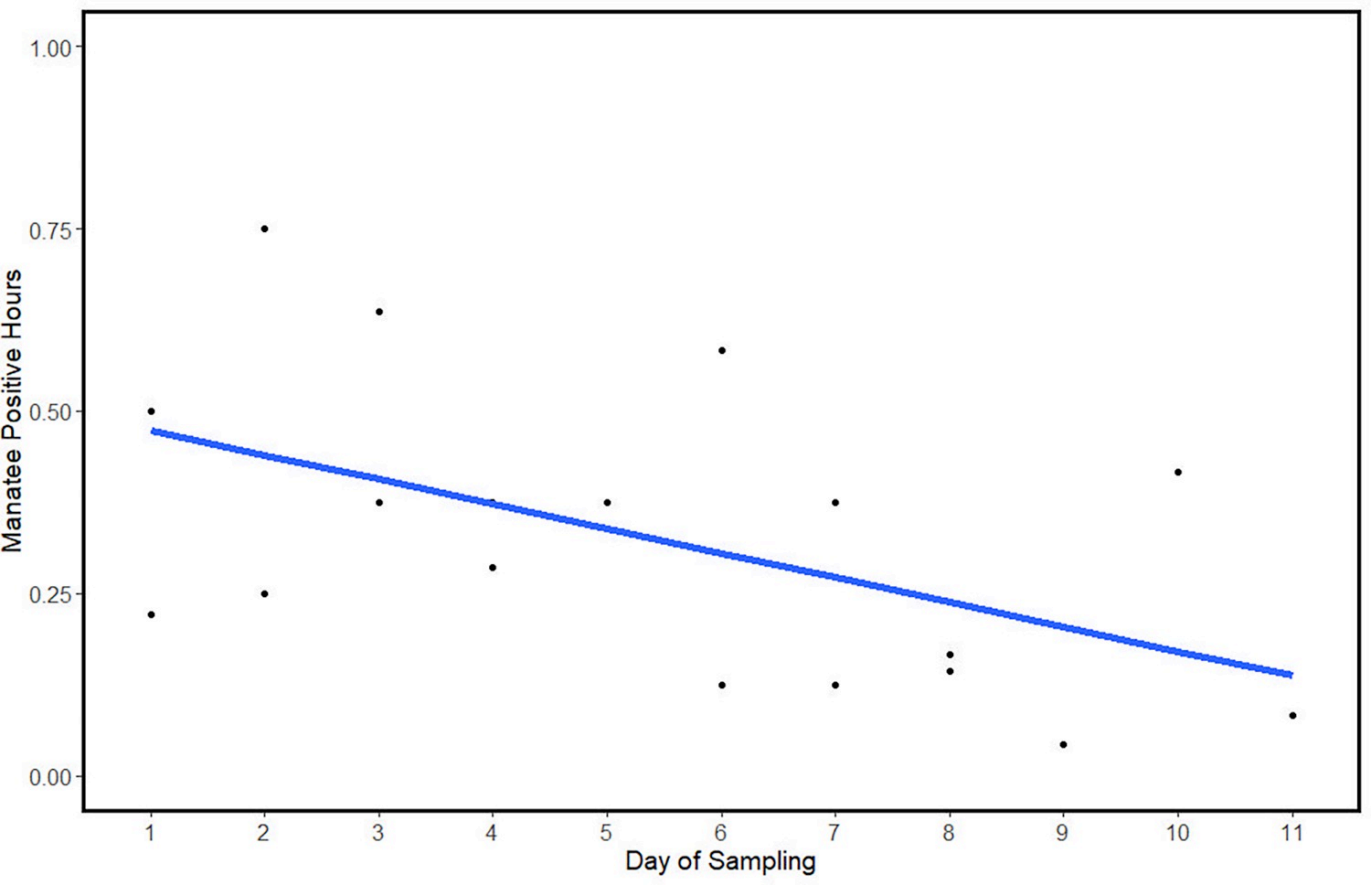
Manatee positive hours (MPH) in relation to the day of sampling. Sampling differed due to timing and the number of days; however, MPH provides an unbiased view of manatee presence across sites. MPH significantly declined over the sampling period regardless of site.

MPH from each site did not violate the assumption of normality (W = 0.95, *p* = 0.395) or homogeneity of variance. Although there was a lower average MPH in the winter (0.297 ± 0.242) than summer (0.368 ± 0.160), the results of the Welch’s two sample t-test determined it was not significant (t(11.349) = 0.720, *p* = 0.486). Since the previous findings suggest that there was no difference between sites, we removed the variable *site* from the model. The best fitting ANOVA (AIC = -7.595) showed that there was a significant difference in MPH due to *day* (F_1,16_ = 6.015, p = 0.026, Fig 5), but not the number of boats (F_1,16_ = 0.533, p = 0.476). The effect size (eta squared, χ^2^) was large for *day* (0.27) and small for the number of boats (0.02). MPH decreased over the course of the study, but the number of boats did not have a significant effect on detection of calls by hour.

**Fig 6:**
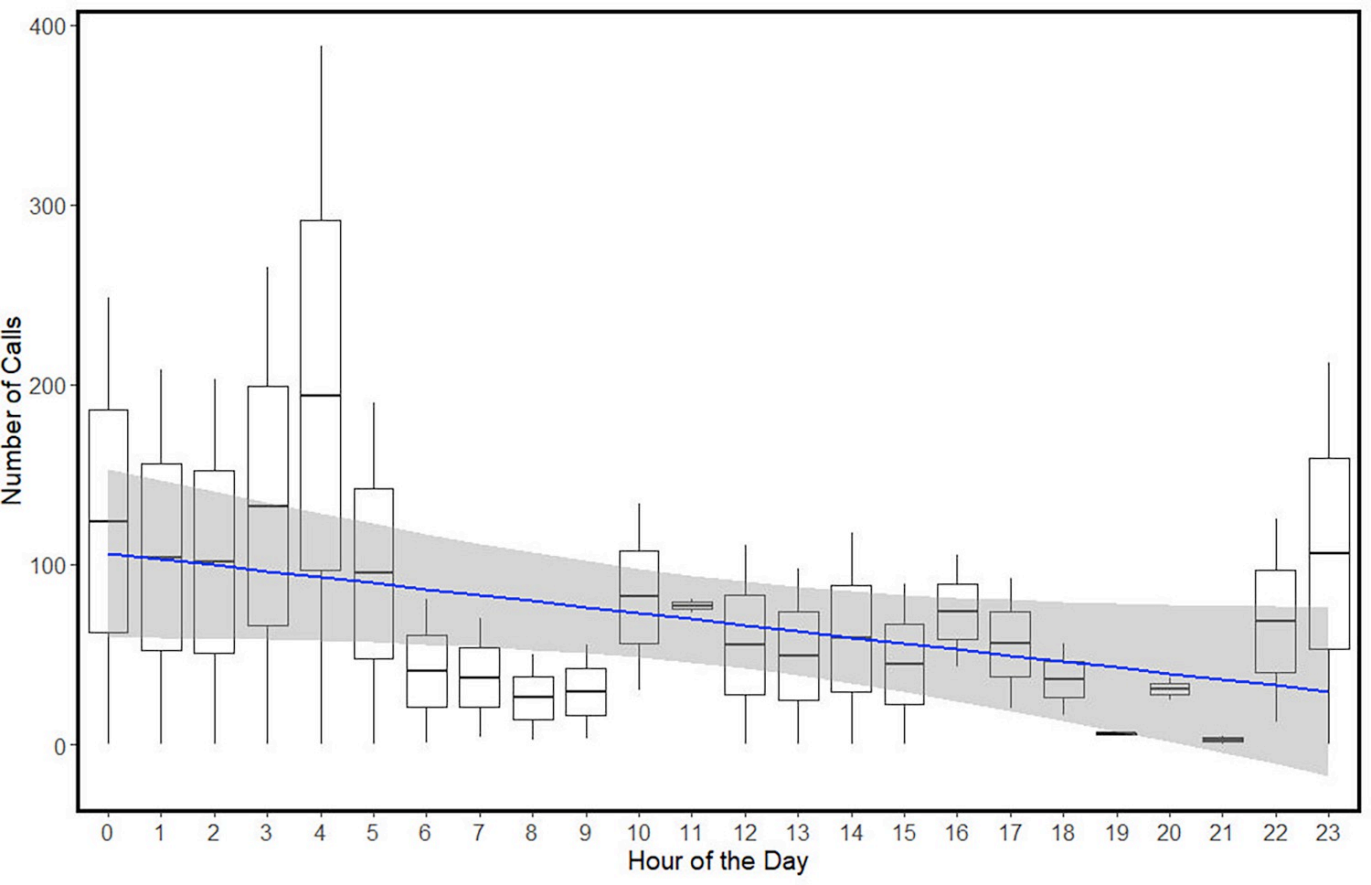
Boxplot depicting the number of manatee calls detected in relation to the hour of the day. Although not significantly different (GLM, blue line with 75% Confidence Interval), the number of calls decreased over the course of the day.

The best fitting model of the GAMM (df = 23.76, AIC = 388.40) determined that there was a difference in the detection of calls due to time of day (χ^2^ = 38.27, *p* < 0.001), the hours after sunset (χ^2^ = 35.62, *p* < 0.001), the day of sampling (z = -4.998, *p* < 0.001), and the interaction of day with the number of boats (z = 2.634, *p* = 0.008) (Fig 6). Call detection increased during daylight and after sunset, decreased over the sampling days, and increased in the presence of boats.

### Diel vocal activity

The number of calls by hour was determined to be non-parametric (Shapiro-Wilk: W = 0.783, *p* < 0.001). A Kruskal-Wallis test used to compare the number of calls per site determined there was no difference in the number of calls due to location (χ^2^ = 33.618, df = 34, *p* = 0.486). Due to these findings and the sampling scheme, data were pooled and then compared by hour of sampling. The best fitting GLM (AIC = 568.74, df = 45) determined that there was not a significant difference in the number of calls due to hour (t = -1.915, *p* = 0.062) or the number of boats (t = 0.528, *p* = 0.599). Although, there does appear to be more calls between 23:00 h and 05:00 h (Figs 3 and 6).

**Fig 7:**
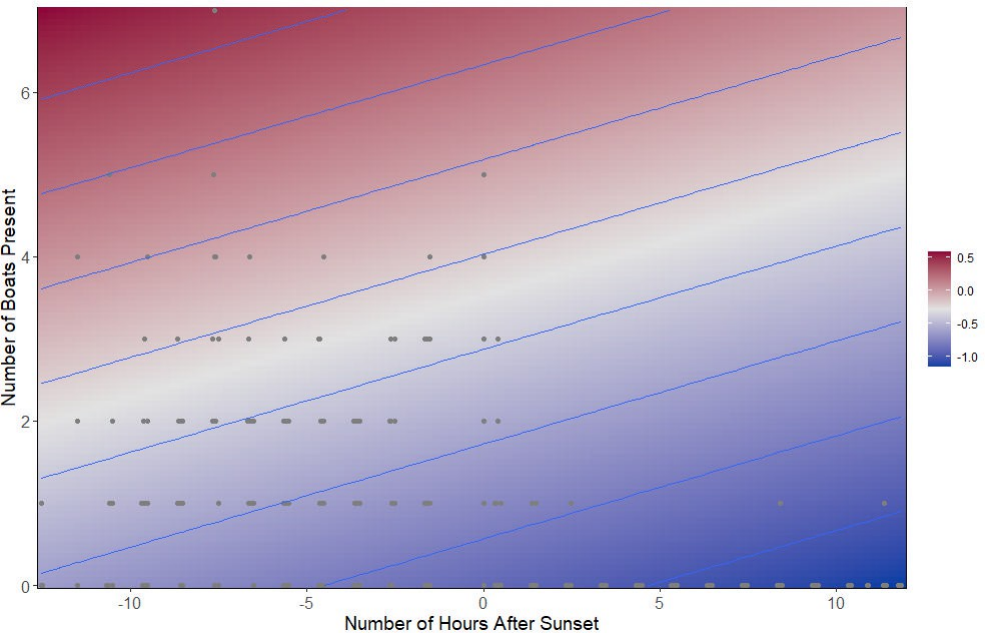
The proportion of tonal calls over all calls in relation to the number of hours after sunset and the number of boats present.

For tonal calls, the best fitting beta regression GAM (df = 4.00, AIC = -15717.73) determined that there was a significant difference in the proportion of tonal calls in relation to the number of boats (*z* = 2.461, *p* = 0.014) and the number of hours after sunset (χ^2^ = 3.898, *p* = 0.049). The proportion of tonal calls decreased after sunset and increased in the presence of boats (Fig 7).

## Discussion

In this study, we explored the temporal trends in acoustic behavior of wild Antillean manatees inhabiting two adjacent microhabitats near St. George’s Caye in Belize. By analyzing vocalizations recorded over 24-hour periods and across multiple days to weeks at both resting hole sites (Sites A and B), we confirm that manatees regularly occupy these habitats. Drone-based photo-identification studies have corroborated these findings, revealing high site fidelity among manatees in the area [48].

Manatees produced vocalizations throughout day and night, but changed their production rates at different times across sites. While manatees are often considered diurnal, our study reveals vocalizations during non-daylight hours as well. This nocturnal activity has also been observed in other sirenian populations, such as dugongs around Talibong Island, Thailand [49], and African manatees (*T*. *inunguis*) in Lake Ossa, Cameroon [50]. Manatees were also found to vocalize during the day, supporting previous visual observations of resting hole use during daylight hours [22]. Overall, our findings suggest that manatees may utilize some resting holes during both day and nighttime hours.

We also observed an increase in manatee vocalizations in conjunction with boat presence. Similar adjustments in vocalization rates have been reported for dolphins (*Tursiops truncatus*) [51] and humpback whales [52] in the presence of small crafts. Boat noise in manatee habitats varies by location and can reduce manatee communication space [53]. Manatees in our study may have compensated for boat presence by increasing vocal effort. However, further investigation is necessary to rule out other factors that could have influenced vocalization rates during the recordings.

Although detections were pooled, Site A exhibited more vocalizations at night, while Site B had more vocalizations during the day. Without simultaneous recordings at each site, it remains unclear if these results stem from daily patterns of movement or seasonal differences. Vocalizations were recorded in two different seasons (site A in the wet season and site B in the dry season), and Antillean manatees are known to exhibit seasonal movement [54]. A prior study by Self-Sullivan et al. (2003) [54] documented the seasonal movement of Belize manatees in this area, specifically between the mainland and the offshore reef. The short distance between sites (~ 1 km) in this study makes it unlikely that seasonal differences affected their vocal behavior. A more plausible explanation is the daily movements of the animals. Site A was smaller in area bordering the caye, and had more calls at night than Site B, which was further from shore and larger but with similar maximum depths. A similar trend was observed with satellite tagged manatees in Puerto Rico. Manatees in Puerto Rico preferred to be closer to shore and in shallower waters during nighttime hours and in deeper waters, further from shore during the day [55]. Future studies should include simultaneous recordings across various habitats to better understand the influence of daily movement patterns on manatee vocal behavior.

Foraging may also play a role in how sites are used. As suggested by Self-Sullivan et al. (2003) [53] seagrass beds surround both sites, providing extensive foraging habitat for manatees. Studies of movements of GPS tagged Antillean manatees indicated they forage at different locations within a small geographic range (4–6 km) [56]. Sites in this study were less than 1 km from each other and vocalizations were recorded at each site, indicating that manatees use both sites. Further, manatees may selectively choose foraging sites based on the type of vegetation. Observational and tracking data showed Antillean manatees in Puerto Rico returned to the same shallow water sites that had abundant *Halodule wrightii* [57]. Although we did not perform a detailed seagrass survey at each site, differences in seagrass composition could influence manatee choice of foraging habitat. One way to determine if manatees prefer one site to the other is to investigate the number of chewing sounds [58] Although this study did not analyze chewing sounds, future studies could incorporate this into the analysis to determine if manatees have feeding preferences based on habitat.

Alternative explanations for the number of detections could be related to the number of individuals, demographic characteristics of the group, and concurrent behavior. The number of manatees visiting each site during the recording period is unknown. Smaller groups of manatees tend to vocalize less [59] than larger groups [60]. Moreover, calves vocalize 2–6 times more frequently than their mothers [36], and it is unclear how many cow-calf pairs visited the both sites. Vocalization rates can also depend on the animals’ behavior; resting manatees vocalize less frequently than those engaged in play [33,36,60]. Further research incorporating more information about the number of individuals in the area and behavior of the manatees can help address these questions to continue to examine connections between diel activity, vocal production, and habitat use.

## Conclusions

Our examination of manatee vocal activity in different microhabitats supports the use of passive acoustic monitoring as an important tool for identifying temporal trends in manatee activity [61]. A deeper understanding of the associations between manatee sound use and activity patterns in relation to habitat and individual life history is needed to contextualize manatee acoustic behavior. These findings may be employed to guide local and national regulations aimed at protecting these vulnerable species. For example, acoustic detections of manatees analyzed in this study were recently used to provide evidence for manatee presence in the region and advocate for the establishment of No Wake Zones to mitigate the effects of boat traffic on manatees in the channels of St. George’s Caye in Belize [38]. Further research is needed to inform the implementation of these zones and other targeted protections based on examinations of manatees’ fine-scale habitat use and activity patterns.

## Supporting information

S1 Table Manatee temporal trends data used in analysis. (XLSX)
